# Modulating the early-life gut microbiota using pro-, pre-, and synbiotics to improve gut health, child development, and growth

**DOI:** 10.1093/nutrit/nuad050

**Published:** 2023-05-11

**Authors:** Benjamin Momo Kadia, Mary Iwaret Otiti, Anouschka S Ramsteijn, Doudou Sow, Babacar Faye, Claire Heffernan, Lindsay J Hall, Joanne P Webster, Alan W Walker, Stephen Allen

**Affiliations:** Department of Clinical Sciences, Liverpool School of Tropical Medicine, Liverpool, United Kingdom; Department of Clinical Sciences, Liverpool School of Tropical Medicine, Liverpool, United Kingdom; Rowett Institute, University of Aberdeen, Aberdeen, United Kingdom; Service de Parasitologie-Mycologie, UFR Sciences de la Santé, Université Gaston Berger, Saint Louis, Senegal; Service de Parasitologie-Mycologie, Faculté de Médecine, Université Cheikh Anta Diop, Dakar, Senegal; London International Development Centre, London, United Kingdom; Intestinal Health, School of Life Sciences, ZIEL—Institute for Food & Health, Technical University of Munich, Freising, Germany; Gut Microbes & Health, Quadram Institute Bioscience, Norwich Research Park, Norwich, United Kingdom; Norwich Medical School, University of East Anglia, Norwich Research Park, Norwich, United Kingdom; Centre for Emerging, Endemic and Exotic Diseases (CEEED), Department of Pathobiology and Population Sciences, Royal Veterinary College, University of London, London, United Kingdom; Rowett Institute, University of Aberdeen, Aberdeen, United Kingdom; Department of Clinical Sciences, Liverpool School of Tropical Medicine, Liverpool, United Kingdom

**Keywords:** environmental enteric dysfunction, gut microbiota, gut pathogens, probiotics, prebiotics, synbiotics

## Abstract

In children exposed to poor hygiene and sanitation, invasion of the gut by pathogenic microbes can result in a subclinical enteropathy termed “environmental enteric dysfunction” (EED) that contributes to undernutrition, growth faltering, and impaired organ development. EED may already be present by age 6–12 weeks; therefore, interventions that can be started early in life, and used alongside breastfeeding, are needed to prevent or ameliorate EED. A healthy gut microbiota is critical for intestinal development and repair, nutrient digestion and absorption, and resisting colonization or overgrowth by pathogens. However, its development can be impaired by several environmental factors. Dietary supplementation with pro-, pre-, or synbiotics may be a pragmatic and safe means of building the resilience of the developing gut microbiota against adverse environmental factors, thereby preventing EED.

## INTRODUCTION

Worldwide, stunting affects 149.2 million and wasting affects 45.4 million of under-5-year-old children, contributing to mortality and loss of human potential.^1^ To date, there have been only limited impacts reported of either nutritional interventions and/or attempts to reduce exposure to infection on malnutrition, resulting in a resurgence of interest in “environmental enteric dysfunction” (EED). EED is characterized by abnormalities of intestinal structure and function and occurs in people exposed to poor sanitation and hygiene.^2^

## HOW DOES ENVIRONMENTAL ENTERIC DYSFUNCTION CONTRIBUTE TO GROWTH FAILURE AND WHAT CAUSES IT?

In children in low- and middle-income countries, enteropathogen invasion of the gut is associated with chronic immune activation that damages the gut mucosa. This damage increases intestinal permeability, which allows pathogens and their products to translocate into the systemic circulation, contributing to systemic inflammation, which inhibits growth hormones and organ development.^2–4^ In addition, shortening of the villi due to chronic inflammation reduces the intestinal surface area, which decreases nutrient digestion and absorption, with possible metabolic pathway derangement.^2^^,^^4^ Environmental enteric dysfunction may already be established by age 6–12 weeks, despite exclusive breastfeeding,^3^^,^^5^ emphasizing the need for early intervention.

## FAILURE OF INTERVENTIONS TO AMELIORATE ENVIRONMENTAL ENTERIC DYSFUNCTION

Complementary feeding, supplementation of the diet with micronutrients (including zinc, vitamin A, and amino acids), probiotics, prebiotics, synbiotics, fatty acids, fish oils, antimicrobials, anti-inflammatory drugs, or combinations of these interventions have had, to date, limited impact on EED (*see*Table S1*in the Supporting Information online*). However, in general, these interventions have been evaluated in older infants and children and, therefore, aimed for the reversal of established EED. To date, there is a paucity of research on interventions aimed instead at preventing EED occurring in the first place.

## THE GUT MICROBIOTA IN EARLY LIFE

Several studies across diverse settings have described similarities in microbiota development in healthy, breastfed infants. Vaginal birth exposes the infant to microbes from the mother’s birth canal, gut, and skin. Shortly after birth, the infant microbiota is transiently dominated by facultative anaerobes such as *Enterobacteriaceae* and *Staphylococcus* species, before being succeeded by microbial communities that are strongly determined by the infant’s diet. In breastfed infants, the main bacteria taxa that replace the initial colonizers are *Bifidobacterium* species, which are exquisitely adapted to growth on human milk oligosaccharides (HMOs) present within breast milk. Following the cessation of breastfeeding and transition to solid foods, by age 2–3 years the composition of the gut microbiota greatly diversifies to that characteristic of the adult microbiota, which is typically dominated by obligately anaerobic bacteria belonging to the *Bacteroidetes* and *Firmicutes* phyla.^6^

**Figure 1:**
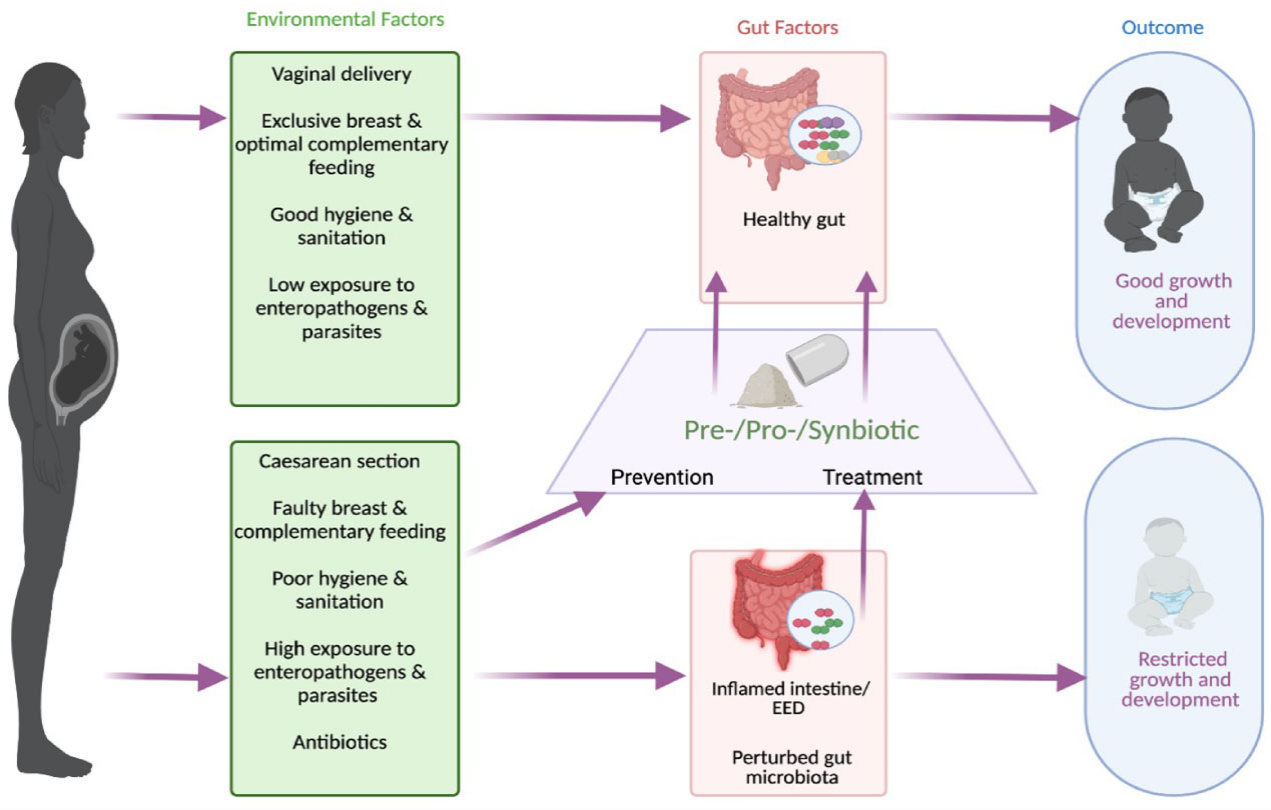
**Pre-, pro-, or synbiotics may prevent or ameliorate environmental enteric dysfunction, improving growth and development (created with Biorender.com).** EED, environmental enteric dysfunction.

The gut microbiota plays critical roles in the formation of gut structure and morphology, mucosal and systemic immunity, and development of metabolic pathways. Of direct relevance to the prevention of EED, nonpathogenic gut microbiota provide “colonization resistance” against invading pathogens.^6^ However, common environmental factors such as caesarean delivery, use of antimicrobials, and parasitic infection can result in perturbed gut microbial communities (Figure 1). Such perturbations are often referred to as “dysbiosis,” although this term is poorly defined; every individual carries a unique collection of gut microbes and there is no specific microbiota composition that is consistently identified as a dysbiotic microbiota.^7^ Nonetheless, there are some features that appear to be more common in “dysbiosis” than in health, such as reduced overall microbial diversity and increased proportional representation of potential opportunistic pathogens such as *Enterobacteriaceae*.^8^ Importantly, a perturbed gut microbiota may not resist colonization of the gut by pathogenic microbes as effectively, or support healthy gut development,^9^ potentially leading to EED and growth failure.

## PRO-, PRE-, AND SYNBIOTICS MAY BUILD THE RESILIENCE OF THE DEVELOPING GUT MICROBIOTA AGAINST ENVIRONMENTAL INSULTS

Probiotics are live, nonpathogenic microorganisms, which, when administered in adequate amounts, can confer a health benefit on the host.^10^ Commonly used probiotics are strains of bifidobacteria and lactobacilli, which may promote and maintain gut health through multiple mechanisms. For example, they may reduce gut inflammation, attenuate increased mucosal permeability following injury, and increase energy harvest from ingested foods.^11^ Critically for the prevention of EED, probiotics may enhance colonization resistance against enteropathogens through the inhibitory effects of short-chain fatty acids and lowering the gut pH, competition for nutrients and attachment points in the gut, secretion of bacteriocins, and stimulation of the mucosal immune system.^12^

Prebiotics are a range of different substrates that are selectively utilized by putatively beneficial indigenous host microorganisms, conferring a health benefit.^13^ Although this term usually applies to dietary ingredients such as oligosaccharides and inulins, the aforementioned HMOs that are present in breast milk can have similar beneficial microbiota-stimulating properties, in particular promoting the growth of bifidobacteria. Mixtures of certain dietary prebiotics, such as short-chain galacto-oligosaccharides and long-chain fructo-oligosaccharides, have been shown to increase bifidobacteria and lactobacilli in the infant gut to levels observed in breastfed infants when added to formula milk.^13^ As well as health benefits resulting from the increased growth of these microbes, prebiotics may also inhibit pathogen growth directly through their antiadhesive properties and serving as decoys for mucosal attachment sites.^13^

Synbiotics combine probiotics with prebiotics, with the aim of enhancing their health benefit. The feasibility, safety, and acceptability of synbiotic administration in newborns at the community level, including those who are exclusively breastfed, was confirmed in a study of over 4500 newborns in rural India where a synbiotic reduced neonatal sepsis.^14^

## RESEARCH CHALLENGES

Despite the clear potential of pre-, pro-, and synbiotics to help in preventing or ameliorating EED, designing clinical trials comes with important challenges. The term “probiotics” encompasses a wide variety of products, with organisms often derived from completely distinct microbial lineages and with very different presumed activities. Given the multiple routes to possible health benefits described above, in order to select specific products for evaluation in clinical trials, greater knowledge is needed to determine whether or not there are “core” benefits that are widespread among probiotic genera or if key mechanistic activities of relevance for EED are limited to specific strains. Probiotic organisms often do not colonize the gut long term, and it is not clear whether or not long-term colonization after administration has discontinued is desirable or necessary in the context of EED. A practical challenge is selecting formulations that ensure the viability of probiotic organisms at point of use, preferably without the need for a cold chain even when used in tropical zones.

With regard to prebiotics, while abundant basic scientific research supports their putatively beneficial effects, evidence from human trials is mixed and inconclusive, and there is a paucity of published research investigating their use in the context of infants at risk of stunting.^15^^,^^16^ An additional problem is that the type of microbial species that respond to a prebiotic intervention depends in large part on the baseline microbiota that is present prior to supplementation. If target organisms are not present in the gut of the consumer, then they clearly will not be promoted by the prebiotic. Human trials must therefore consider the highly individual nature of microbiota responses that may result from prebiotic stimulation, which are not easily predicted a priori.^17^ In addition, alongside effects on the microbiota, prebiotic ingredients can interact directly with the intestinal epithelium and immune system.^16^ Consequently, the effects of different prebiotics are not expected to be equivalent and it is not yet understood which may be the most appropriate for consumption by a given target population.

With synbiotics, it is not clear whether an optimal product would include a prebiotic that is a nutrient source for the included probiotics in addition to its effects on the overall growth and metabolism of the indigenous gut microbiota. Furthermore, although these products have a well-established safety record, close monitoring for adverse effects during clinical trials is critical. Finally, trials should include measurement of gut health and systemic inflammation as important outcomes, as pre-, pro-, or synbiotics may ameliorate these underlying pathological processes that impair the development of the brain and other organs, and which may occur independently of effects on weight and height gain.^18^

## CONCLUSIONS

Malnutrition continues to underlie much childhood mortality and loss of human potential. Interventions that improve gut health may add to current preventative measures and also bring important benefits independent of growth. Pro-, pre-, and synbiotic administration may be a pragmatic and safe means of boosting the resilience of the developing gut microbiota against adverse environmental influences. This emerging area of research has the potential to inform global policies related to child malnutrition and development.

## Supplementary Material

nuad050_Supplementary_Data

## Supporting Information

The following Supporting Information is available through the online version of this article at the publisher’s website.

*Table S1* **Interventions that have been trialed previously to prevent or ameliorate environmental enteric dysfunction**
