# Supplementary material for: Modulating the early-life gut microbiota using pro-, pre-, and synbiotics to improve gut health, child development, and growth
Source: Nutr Rev. 2023 May 11;82(2):244–7. doi: 10.1093/nutrit/nuad050 (PMC10777666; doi:10.1093/nutrit/nuad050)
Supplement: nuad050_Supplementary_Data [file nuad050_supplementary_data.docx]

**Supplementary table 1: Interventions to prevent or ameliorate environmental enteric dysfunction (EED)**

| **Intervention** | **Target group** | **Main findings** | **Reference** |
| --- | --- | --- | --- |
| Probiotics: *Lactobacillus rhamnosus* GG for 30 days | 161 healthy Malawian children age 3-5 years | No effect on dual sugar permeability test | Galpin L et al. Effect of Lactobacillus GG on intestinal integrity in Malawian children at risk of tropical enteropathy. Am J Clin Nutr 2005; 82(5): 1040-5. |
| High dose vitamin A compared to standard dose | 197 mother/infant dyads; mother at delivery and infants from age 2 months | No effects on *Helicobacter pylori* infection, infant pneumococcal carriage or dual sugar permeability test | Darboe MK et al. Effectiveness of an early supplementation scheme of high-dose vitamin A versus standard WHO protocol in Gambian mothers and infants: a randomised controlled trial. Lancet 2007 Jun 23;369(9579):2088-96. |
| Alanyl-glutamine for 10 days | 107 Brazilian children with mild undernutrition age 6m–8 years | Statistically significant decreased lactulose excretion but minor effect. | Lima NL et al. Wasting and intestinal barrier function in children taking alanyl-glutamine-supplemented enteral formula. J Pediatr Gastroenterol Nutr 2007; 44(3): 365-74. |
| Non-absorbable antibiotic: Rifaximin for 7 days | 144 healthy Malawian children age 3-5 years | No effect on dual sugar permeability test | Trehan I et al. A randomized, double-blind, placebo-controlled trial of rifaximin, a nonabsorbable antibiotic, in the treatment of tropical enteropathy. Am J Gastroenterol 2009; 104(9): 2326-33. |
| LCPUFA from age 3-9 months | 172 rural Gambian infants age 3–9 months. | Modest effect on MUAC. No effects on other anthropometric measures, dual sugar permeability test, faecal calprotectin, daily morbidity, cognitive development | van der Merwe LF et al. Long-chain pufa supplementation in rural African infants: A randomized controlled trial of effects on gut integrity, growth, and cognitive development. Am J Clin Nutr 2013; 97(1): 45-57. |
| Zinc/albendazole: single dose albendazole or zinc sulfate for 14 days | 222 asymptomatic Malawian children age 1-3 years | Increase in dual sugar permeability test occurred in all 3 groups, but statistically significantly less in the intervention groups than the control group. | Ryan KN et al. Zinc or albendazole attenuates the progression of environmental enteropathy: A randomized controlled trial. Clin Gastroenterol Hepatol 2014; 12(9): 1507-1513. |
| MMN with or without fish oils for 24 weeks | 230 Malawian children aged 12–35 months | Statistically significant but minor effects on dual sugar permeability test. No effect on length gain. | Smith HE et al. Multiple micronutrient supplementation transiently ameliorates environmental enteropathy in Malawian children aged 12-35 months in a randomized controlled clinical trial. J Nutr 2014; 144(12): 2059- 2065. |
| Prebiotic: resistant starch for 2 weeks | 18 stunted Malawian children age 3-5 years | Change in faecal microbiota and SCFAs. No reduction in faecal calprotectin. | Ordiz MI, May TD, Mihindukulasuriya K, et al. The effect of dietary resistant starch type 2 on the microbiota and markers of gut inflammation in rural Malawi children. Microbiome 2015; 3:37. |
| MMN for 24 weeks and 2 doses of Zn and albendazole | 254 rural Malawian children age 12-35 months | No effect on dual sugar permeability test or growth | Wang AZ et al. A combined intervention of zinc, multiple micronutrients, and albendazole does not ameliorate environmental enteric dysfunction or stunting in rural Malawian children in a double-blind randomized controlled trial. J Nutr 2017; 147: 97-103. |
| Complementary feeding with cowpea | 291 Malawian infants age 6 months | Less fall in LAZ score 6-9 months. No effect on lactulose excretion. | Stephenson KB et al. Complementary feeding with cowpea reduces growth faltering in rural Malawian infants: a blind, randomized controlled clinical trial. Am J Clin Nutr 2017 Nov 1. pii: ajcn160986. |
| Water, sanitation and hygiene; improved feeding | 1169 rural Zimbabwean infants age 0-18 months | No effect on biomarkers of EED | Gough EK, et al. Effects of improved water, sanitation, and hygiene and improved complementary feeding on environmental enteric dysfunction in children in rural Zimbabwe: A cluster-randomized controlled rial. PLoS Negl Trop Dis 2020; 14(2): e0007963 |
| Microbiota-Directed Food Intervention | 118 slum-dwelling Bangladeshi  children age 12-18 months with moderate acute malnutrition | Improved gain in WLZ and WAZ; change in faecal microbiota; increased levels of proteins mediating bone growth and neurodevelopment. | Chen RY, et al. A Microbiota-Directed Food Intervention for Undernourished Children. N Engl J Med. 2021 Apr 22;384(16):1517-1528. |

Notes: LCPUFA= long chain polyunsaturated fatty acids; MUAC = mid-upper arm circumference; MMN = multiple micronutrients; SCFA = short-chain fatty acids; LAZ = length-for-age z score; WLZ = weight-for-length z score; WAZ = weight-for-age z score
